# Non-invasive prediction of invasive lung adenocarcinoma and high-risk histopathological characteristics in resectable early-stage adenocarcinoma by [18F]FDG PET/CT radiomics-based machine learning models: a prospective cohort study

**DOI:** 10.1097/JS9.0000000000003566

**Published:** 2025-09-30

**Authors:** Man Sun, Huan Zhou, Jun Chen

**Affiliations:** Department of Oncology, The Second Hospital of Dalian Medical University, Shahekou District, Dalian, Liaoning, China

*Dear Editor,*

We read with interest the recent article by Cao *et al*^[1]^, who developed [18 F]FDG PET/CT-based AutoML radiomics models to preoperatively distinguish invasive adenocarcinoma (IA) from AIS/MIA and to predict high-risk histopathologic features in early-stage lung adenocarcinoma. As image-informed surgical decision-making gains momentum – particularly for subcentimeter peripheral nodules – the study represents a timely contribution to precision thoracic surgery. However, to evaluate the clinical readiness of such tools, several conceptual and translational limitations deserve further scrutiny. This study complies with the TITAN 2025 guidelines for transparent and ethical reporting of AI-assisted research^[2]^.

First, despite impressive AUCs (0.93 for IA and 0.85 for high-risk pathology), the model’s misclassification patterns remain unexplored. Beyond accuracy, clinical deployment demands case-level error mapping^[3]^. Without detailed analysis of false positives and negatives, surgeons cannot determine when to rely on or override predictions. Overestimating AIS/MIA may lead to overtreatment, while underestimating IA risks undertreatment. We recommend incorporating explicit audits – such as heatmaps, subgroup profiles, or illustrative vignettes – to clarify failure modes and delineate the model’s risk boundaries.

Second, although the AutoML models performed well, the authors did not report model architecture, feature contributions, or interpretability of key radiomic features like GLCM entropy. Opaque algorithms limit clinical trust, informed consent, and regulatory approval. In the surgical setting, interpretability is not optional; it underpins clinical trust and ethical deployment^[4]^. Moreover, international regulatory frameworks, such as the EU AI Act and FDA SaMD, increasingly mandate transparency^[5]^. Future studies should disclose model composition, quantify feature importance, and assess biological plausibility to ensure clinical utility.

Third, the model relies solely on single-timepoint PET/CT, overlooking the temporal evolution of lung adenocarcinoma. Static features fail to capture dynamic traits – such as growth kinetics or metabolic acceleration – that better reflect biological aggressiveness. As early-stage lesions often evolve from AIS/MIA to IA, omitting temporal signals risks misclassification^[6]^. We advocate for time-aware modeling strategies, using kinetic imaging data or recurrent architectures, to enhance biological relevance and surgical applicability. Such dynamic indicators could directly inform decisions regarding segmentectomy versus lobectomy or guide the extent of lymph node dissection in surgical planning.

Finally, using postoperative histology as the reference standard introduces a paradox, as such labels are unavailable at the time of surgical planning. Since operative decisions rely on preoperative imaging and intraoperative findings, validating against retrospective pathology risks performance inflation and workflow misalignment^[7]^. Future studies should adopt pragmatic endpoints – such as resection choice or lymphadenectomy extent – to better reflect real-world decision-making and enhance translational robustness.

While Cao et al. contribute valuably to PET-based radiomics, critical translational gaps remain – particularly regarding decision alignment, interpretability, and biological relevance. To realize clinical potential, radiomics must shift from retrospective metrics to transparent, dynamic, and actionable frameworks. This correspondence aims to catalyze that shift by identifying barriers to clinical integration and encouraging development of models that are not only accurate but also trustworthy and seamlessly compatible with surgical workflows – especially when embedded into preoperative pathways involving multidisciplinary team discussions, intraoperative navigation, and patient counseling.

## Data Availability

No datasets were generated or analyzed for this article. Data sharing is not applicable.

## Research registration unique identifying number (UIN)

This study did not involve human participants, and therefore, registration and a Unique Identifying Number (UIN) were not required.
